# Evaluating the Impact of the Size of PbS Colloidal Quantum Dots on Photodetection Performance

**DOI:** 10.3390/nano16171073

**Published:** 2026-08-28

**Authors:** Freddy Garcia, Suraj Patel, Anthony Monterrosas, John Navarro, Seungbae Ahn, Oscar Vazquez-Mena

**Affiliations:** 1 Aiiso Yufeng Li Family Department of Chemical and Nano Engineering, Center for Memory and Recording Research, University of California San Diego, La Jolla, CA 92093, USA; f8garcia@ucsd.edu (F.G.); sup027@ucsd.edu (S.P.); amonterrosasmendoza@ucsd.edu (A.M.); jon016@ucsd.edu (J.N.); 2 Materials Science and Engineering Program, University of California San Diego, La Jolla, CA 92093, USA; 3 Division of Electronics and Electrical Information Engineering, Korea Maritime and Ocean University, Busan 49112, Republic of Korea

**Keywords:** colloidal quantum dots, photodetectors, graphene, quantum dot film mobility, size effect

## Abstract

Colloidal quantum dots (CQDs) provide size-tunable optoelectronic properties, enabling broadband photodetection from the visible to the short-wave infrared (SWIR). However, CQD photodetector performance depends on different processes such as optical absorption, charge transport, and photogain mechanisms in CQD solids. Here, we investigate the impact of PbS CQD size on the electrical conductivity and photodetection performance of CQD films and hybrid graphene/CQD photodetectors. Three CQD sizes are studied: d~3.06 nm, d~3.78 nm, and d~5.27 nm, with exciton peaks at λ_e_~935 nm, λ_e_~1080 nm, and λ_e_~1550 nm, respectively. Electrical measurements show that the largest CQDs exhibit higher conductivity. In contrast, photodetection measurements reveal that the largest-sized CQDs (~5.27 nm) produce the lowest photoresponse, both as bare CQDs and as hybrid graphene/CQD photodetectors. Spectral and power-dependent measurements show decreasing responsivity with increasing optical power, consistent with trap-mediated photoconductive gain. These results indicate that the CQD size can have a significant effect on the optoelectronic performance of CQD devices, requiring materials, interfaces, and design optimization to maintain high photodetector performance.

## 1. Introduction

Colloidal quantum dots (CQDs) are semiconductor nanocrystals whose quantum confinement enables strong, size-tunable optoelectronic properties [1,2,3,4,5]. Their direct and tunable bandgap supports efficient light absorption and photocarrier generation, making them attractive for photodetectors [6,7,8,9,10,11], displays [12,13,14], and solar cells [15,16,17]. In addition, CQDs offer a wide spectral range of operation—from the ultraviolet to the short-wave infrared, depending on composition and size—and exhibit good chemical stability when properly surface-passivated and encapsulated. Their solution-based processing further provides a pathway to scalable and low-cost manufacturing. As a result, CQDs have been explored across telecommunications, flexible electronics, neuromorphic devices, sensing, energy harvesting, and biomedical applications. CQDs have also been used in food safety monitoring [18]. Lead sulfide (PbS) CQDs are particularly relevant for infrared optoelectronics, with absorption and emission spanning the near-infrared (NIR) to short-wave infrared (SWIR) range (λ ≈ 850–1800 nm) [19,20,21]. This spectral coverage positions them as cost-effective alternatives to conventional but high-cost materials such as Germanium and Indium Gallium Arsenide [22]. Despite these advantages, CQD solids exhibit intrinsically limited charge transport. Carrier motion occurs via thermally activated hopping between nanocrystals, resulting in lower conductivity compared to bulk semiconductors [23,24,25]. The interface between CQDs is strongly dependent on particle size, surface passivation, and ligand chemistry, resulting in challenges for optimizing CQD device performance [26]. The CQD boundaries and surface also provide a large number of trapping states that result in high photogain, which also enhances photoresponsivity, whereas surface passivation, hybridization, and hybrid structures can facilitate charge transfer and reduce detrimental trapping and recombination [27,28]. While tuning CQD size enables continuous tuning of the spectral response, it also affects interparticle spacing and boundaries, which can significantly impact charge transport. Consequently, optimizing CQD-based devices requires balancing spectral tunability with electronic performance. In this work, we investigate the size-dependent performance of PbS CQD films, focusing on electrical conductivity and photodetection characteristics in both bare CQD and hybrid graphene–CQD (Gr/CQD) photodetector architectures and exploring the impact of CQD size on device performance.

**Figure 1:**
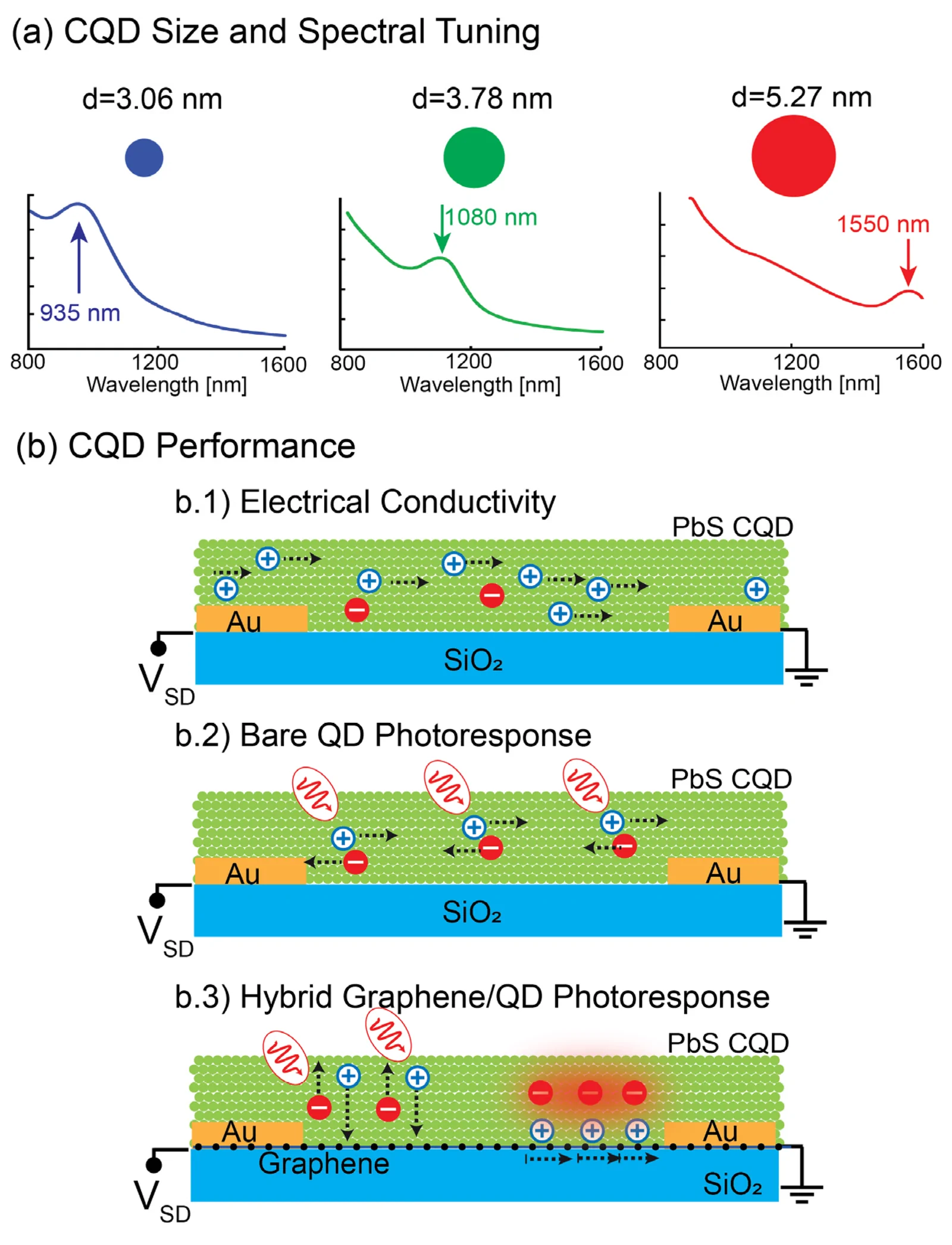
Size, spectral tuning, and optoelectronic performance. (**a**) Size tuning of colloidal quantum dots enables spectral tuning of their bandgap, showing how, as the CQDs increase their size, the exciton peak is red-shifted. (**b**) Analysis of the electrical conductivity (**b.1**), bare CQD lateral detector photoresponse (**b.2**), and photoresponse in a hybrid graphene/CQD photodetector architecture (**b.3**).

Herein, we investigate the performance of three types of PbS CQDs with different sizes, focusing on their electrical conductivity and photodetection performance. Figure 1 shows a schematic image of our study. Figure 1a illustrates the CQDs with different sizes leading to different bandgaps and exciton peaks. In this study, our PbS CQDs have sizes (d) and exciton peaks (λ_e_) of d~3.06 nm and λ_e_~935 nm, d~3.78 nm and λ_e_~1080 nm, and d~5.27 nm and λ_e_~1550 nm, showing the expected red-shift as the CQD size increases. We begin our study by characterizing the CQD morphology and optical properties, including absorption coefficients and bandgaps extracted from Tauc plot analysis. We then perform three experiments shown in Figure 1b to evaluate how the CQD size impacts their optoelectronic performance. First, we analyze the electrical conductivity as a function of CQD size to study the impact of CQD size on charge transport as shown in Figure 1b.1. Second, we study the performance of bare CQD lateral photodetectors as shown in Figure 1b.2. Finally, we investigate the performance of CQDs in a hybrid graphene–CQD (Gr/CQD) photodetector architecture as shown in Figure 1b.3. In this case, the CQD film serves as a light absorber through which photocarriers are transferred to graphene, which in turn acts as the charge transport channel [9,10,29,30,31]. Together, these measurements provide a comprehensive assessment of charge transport and photoresponse across different CQD sizes, offering direct insight into how size-dependent spectral tuning influences the optoelectronic performance of PbS CQD-based devices.

## 2. Materials and Methods

CQD synthesis and size tuning: 0.94 g of lead oxide (PbO) was dissolved in 25 mL of 1-octadecene (ODE) with different amounts of oleic acid. The components were mixed in a three-neck flask to achieve various sizes of PbS CQDs [31]. After that, the mixture was degassed under vacuum at 90 °C for one hour until dissolved. When the color of the solution became clear, 420 µL of bis(trimethylsilyl) sulfide dissolved in 12.8 mL of ODE was injected into the solution. After 30 s, the flask was cooled down in water. The PbS CQDs were separated from the raw solution by centrifugation, followed by cleaning with toluene and acetone, and then dissolved in toluene. The PbS CQD solution was filtered with a 0.25 µm pore size filter. The oleic acid volume and the corresponding obtained exciton peaks are: 2.98 mL for λ_e_~935 nm, 11.92 mL for λ_e_~1080 nm, and 35.76 mL for λ_e_~1550 nm.

CQD characterization: The optical absorption of the CQD films was measured by UV-Vis spectroscopy (UH4150, Hitachi, Japan). The PbS CQDs were imaged by transmission electron microscopy (TEM). The particle size was analyzed using ImageJ 1.54g software.

**Figure 2:**
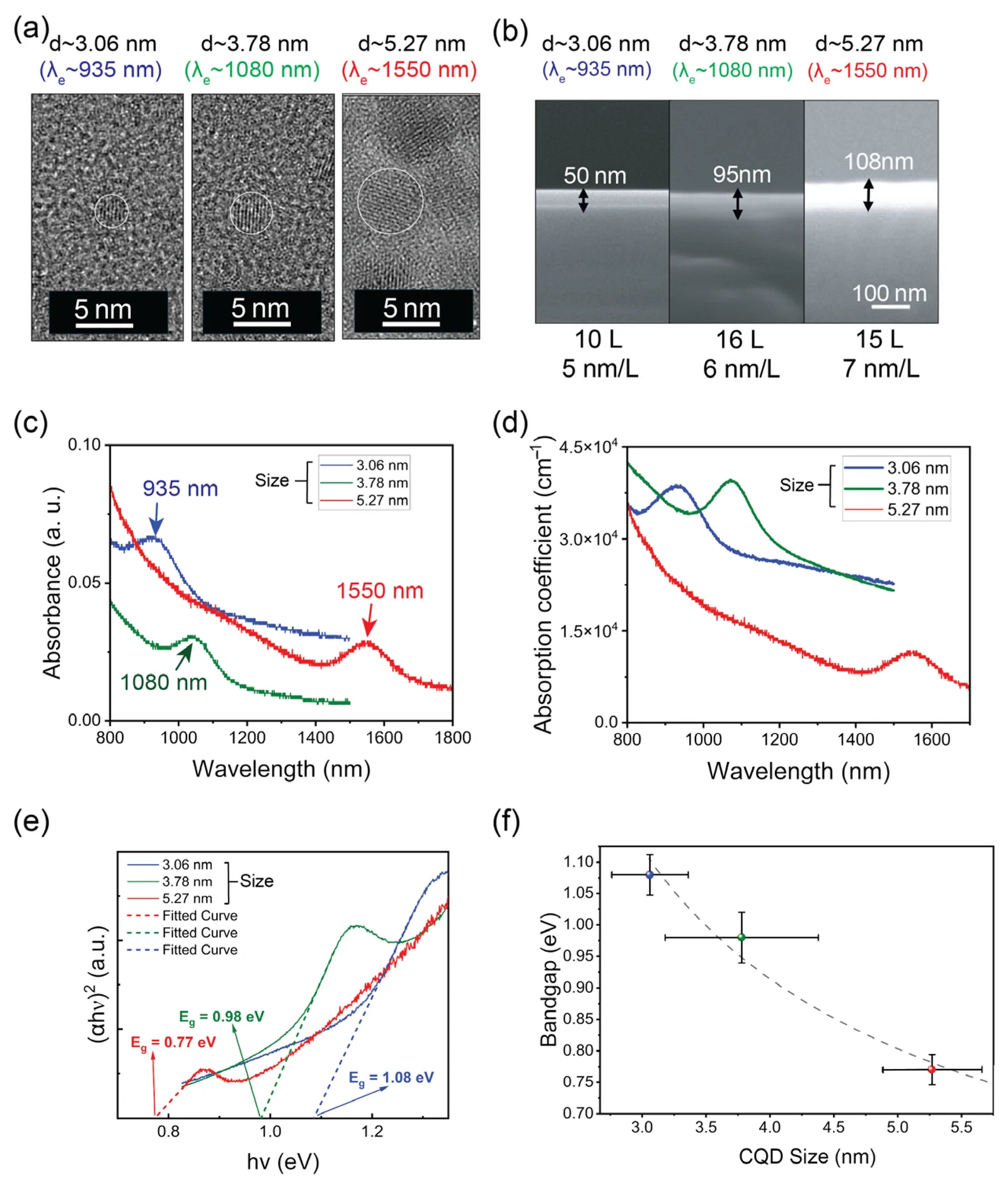
Nanoparticle size, thickness, and optical properties of CQD films. (**a**) TEM image of the CQDs for this study showing single-crystal structure. After statistical analysis, the average sizes obtained were d~3.06 ± 0.33 nm, d~3.78 ± 0.39 nm, and d~5.27 ± 0.59 nm. (**b**) SEM cross sections of the corresponding CQD films used for thickness calibration. The thickness (t) for each case is t = 50 nm, 95 nm, and 108 nm. The thickness per layer t_L_ for each CQD type is t_L_~5 nm/layer for d~3.06 nm, t_L_~6 nm/layer for d~3.78 nm, and t_L_~7 nm/layer for d~5.27 nm. (**c**) Measured absorption spectra of CQD films on glass, showing exciton peaks at λ_e_~935 nm for d~3.06 nm, λ_e_~1080 nm for d~3.78 nm, and λ_e_~1550 nm for d~5.27 nm. (**d**) Calculated absorption coefficient as a function of wavelength. (**e**) Tauc plots and linear extrapolations to estimate the bandgap of the CQD films. (**f**) Bandgap as function of CQD size, showing the expected decrease in bandgap for larger CQDs. The dashed line corresponds to a 1/R^2^ fitting characteristic of quantum confinement.

Device fabrication: For the electrical conductivity and photoresponse of bare PbS CQDs, the films were prepared by a spin-coating method. All devices were fabricated on silicon chips with a 300 nm thick thermal silicon oxide layer (University Wafers, Silicon Resistivity: 0.001–0.005 Ohm-cm) and prepatterned Au/Cr contacts (Au: 50 nm thick, Cr: 5 nm thick). A solution of 0.045 mL of PbS quantum dots in toluene was deposited on the substrate by spin coating at 2500 rpm for 10 s. Next, 0.03 M tetrabutylammonium iodide (TBAI) solution in methanol was added for ligand exchange by incubation for 30 s, followed by cleaning in methanol. The concentration of PbS CQD solutions for spin coatings was 19 mg/mL for λ_e_~935 nm CQDs, 15 mg/mL for λ_e_~1080 nm CQDs, and 20 mg/mL for λ_e_~1550 nm CQDs. These concentrations were obtained from the synthesis process and QD solution washing. For Gr/CQD photodetectors, CQDs were deposited on graphene layers on Si/SiO_2_ (SiO_2_ 300 nm thick) chips with prepatterned Au/Cr electrodes as well. The graphene monolayers on copper (Graphenea, Spain) were transferred by wet transfer using PMMA as a supporting layer and using ammonium persulfate for copper etching. After the graphene transfer, PMMA was removed with acetone and isopropanol. After PMMA removal, CQDs were spin-coated as described before. For all devices, conductivity measurements, bare CQDs, and Gr/CQD devices, we prepared CQD films with a thickness of ~100 nm for each CQD size. In order to obtain 100 nm thick films for different solutions of CQDs of different sizes, we calibrated the thickness per layer using scanning electron microscopy to image cross sections of the films and estimate the thickness per layer (Figure 1b). For the λ_e_~935 nm CQDs, 20 layers were spin-coated; for the λ_e_~1080 nm CQDs, 17 layers were spin-coated; and finally, for the λ_e_~1550 nm CQDs, 14 layers were spin-coated. The number of layers was based on the thickness per layer calibration from Figure 2b in order to obtain a thickness of 100 nm for each of the CQD layers of different CQD sizes.

Electrical and photoresponse measurements: Electrical current under bias voltage was measured using a Keithley (Cleveland, OH, USA) 2400 source meter. The light sources were laser diodes from Thorlabs with different wavelengths: 450 nm laser diode (CPS450), 532 nm laser diode (CPS532), and 635 nm laser diode (CPS635R). Current versus voltage and current versus time data under laser illumination were measured using a Keithley 2400 source meter. Spectral response was measured using a Keithley 2400 source meter under a xenon lamp with a monochromator (CS260-RG-3-FH-D, Newport).

## 3. Results

The size and optical properties of the PbS CQDs for this study are shown in Figure 2. Figure 2a shows three panels with transmission electron micrographs of CQDs. After statistical analysis of the TEM images, the average sizes obtained were d~3.06 ± 0.54 nm, d~3.78 ± 0.67 nm, and d~5.27 ± 0.98 nm. Supplementary Information Section S1 shows low-magnification TEM images and histograms confirming a narrow and reproducible size distribution consistent with the size-tunable, monodisperse synthesis reported here. The TEM images show the single crystal nature of the CQDs. Supplementary Information Section S1 also shows digital FFT analysis showing sharp symmetric spots, indicating one crystalline domain per particle. Figure 2b shows corresponding cross sections of films formed by spin coating of their respective type of CQDs used to estimate the thickness obtained per CQD layer. The thickness (t) and number of spin-coated layers (L) for each film are t~50 nm for 10 L, t~95 nm for 16 L, and t~108 nm for 15 L. The details of the spin-coating protocol are described in the Section 2. From the film thickness and number of layers, we can estimate that for our particular film deposition conditions, each layer produces a thickness per layer (t_L_) of t_L_~5 nm/layer for d~3.06 nm, t_L_~6 nm/layer for d~3.78 nm, and t_L_~7 nm/layer for d~5.27 nm. This parameter t_L_ is important to achieve the desired thickness based on the number of spin-coated layers L. Atomic Force Microscopy (AFM) scans and roughness analysis show solid films with roughness below 10% of the film thickness, without major cavities or voids. The roughness estimations from AFM analysis are R_z_~4.755 nm for CQD size of 3.06 nm, R_z_~4.311 nm for CQD size of 3.78 nm, and R_z_~7.895 nm for QD size of 5.27 nm. The AFM images and line profiles are shown in the Supplementary Information Section S2. The experimental optical absorption spectra of the CQD films are shown in Figure 2c, showing the clear red-shift in the exciton peak as the size of the CQD increases. The exciton peaks λ_e_ are located at λ_e_~935 nm for d~3.06 nm, λ_e_~1080 nm for d~3.78 nm, and λ_e_~1550 nm for d~5.27 nm. By taking optical spectra of films of different thicknesses and fitting the transmission vs thickness to an exponential using the Beer-Lambert law (T = T_0_exp(–αt)), we extracted the absorption coefficient (α) as a function of wavelength for each of the three CQDs under study. The plots of α as a function of wavelength (λ~800–1800 nm) are shown in Figure 2d. The absorption coefficients for the extended range of λ~500–1800 nm are shown in Supplementary Information Section S3. From the absorption coefficients, it is possible to produce Tauc plots by plotting (αhν)^n^ vs. hν, where α is the absorption coefficient, h is Planck’s constant, ν is the frequency, and n = 2 for the direct bandgap of the CQD. By extrapolating the Tauc plots to the x-axis, we can extract the bandgaps, which correspond to E_g_~1.08 eV for d~3.06 nm, E_g_~0.98 eV for d~3.78 nm, and E_g_~0.77 eV for d~5.27 nm, as shown in Figure 2e. Finally, the bandgaps are plotted as a function of CQD size in Figure 2f. The horizontal error bars correspond to size variations extracted from Image J size analysis, and the vertical error bars come from errors extracted from Tauc plot bandgap energy analysis. The dashed line corresponds to a 1/R^2^ fitting, which is typical for quantum confinement. Overall, the CQD films show an exciton peak, characteristic of direct bandgap materials, in the NIR range. The absorption coefficient of the CQDs is on the order of 10^4^ 1/cm, characteristic also of similar PbS CQD films reported in the literature [32].

**Figure 3:**
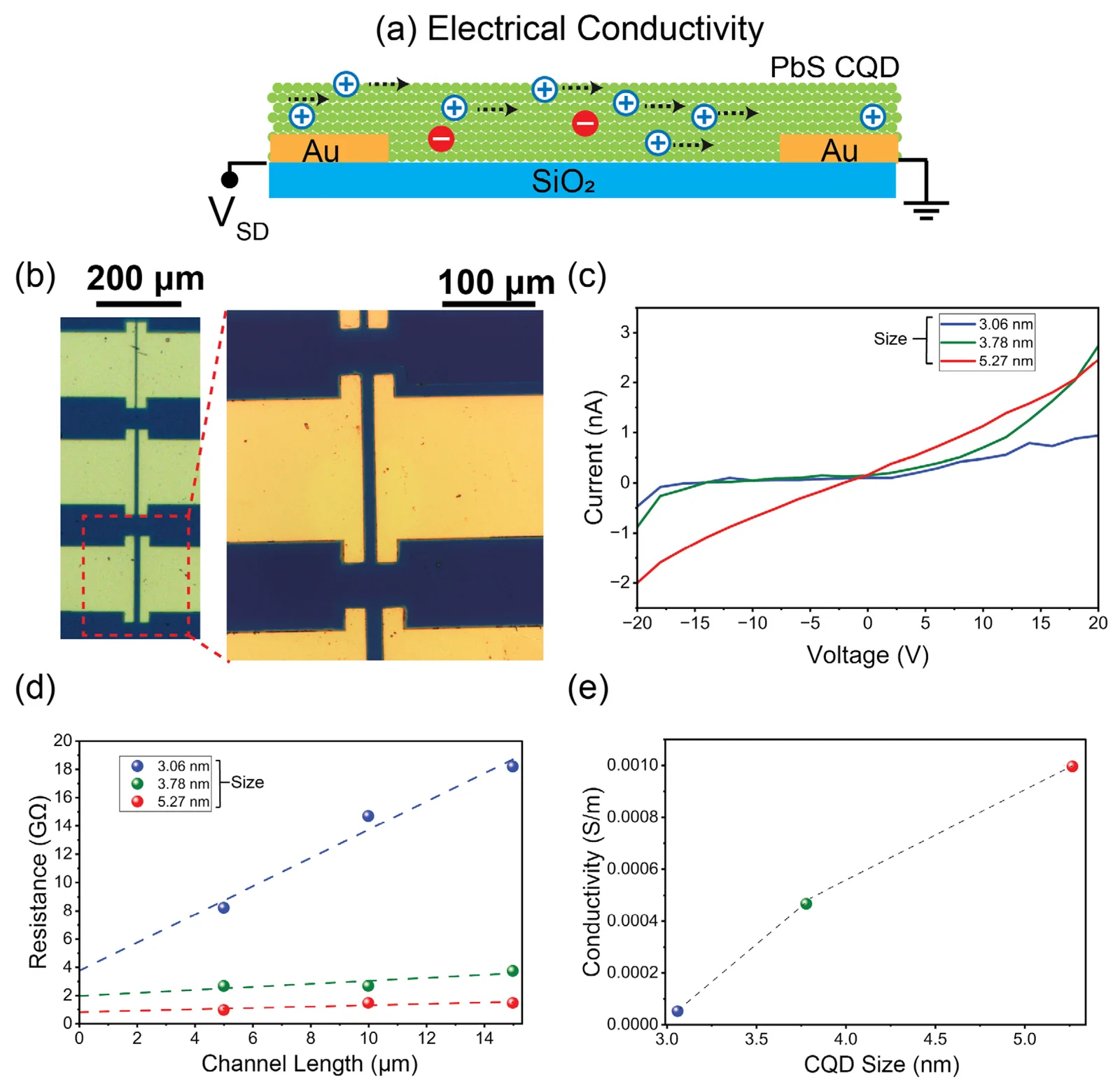
Electrical conductivity of CQD films. (**a**) Schematics of electrical conductance measurements on the CQD films, dominated by majority carrier transport. (**b**) Optical micrograph of a typical CQD film device. The electrical contacts are made of Au/Cr. The channel width is 200 μm, and the lengths are 5, 10, and 15 μm. (**c**) I/V curves of CQD films with bias voltage V_SD_ from −20 to 20 V. (**d**) Electrical resistance as function of channel length for the three different CQD sizes, showing the expected increase in resistance as the channel length increases. (**e**) Conductivity extracted from panel (**d**) as function of CQD size, showing a clear trend of increasing conductivity as the CQD size increases.

The electrical conduction characteristics of the CQDs are shown in Figure 3. The main conduction mechanism is the transport of majority p-type carriers characteristic of PbS CQD films, as shown in Figure 3a. CQD films 100 nm thick were deposited on silicon substrates 500 μm thick with a thermal silicon oxide top layer 300 nm thick and pre-defined Au/Cr (100 nm/10 nm thick) contacts deposited by e-beam evaporation and lift-off. Figure 3b shows an optical micrograph of the tested device. The contacts have channel lengths (*l*) of 5, 10, and 15 μm, and channel widths (W) of 200 μm. Figure 3c shows the current–voltage (I–V) characteristics of the PbS CQD films for the three sizes in dark conditions. While the response is approximately linear at low bias, all devices exhibit a clear non-linear increase in current beyond ~10 V, indicating field-dependent transport. This behavior is consistent with hopping conduction in CQD solids, where higher electric fields enhance carrier injection and inter-dot tunneling, leading to non-linear current [33,34,35]. Figure 3c also shows that the largest CQDs with a size of (d~5.27 nm) have the highest conductance. Figure 3d shows the electrical resistance R_e_ as a function of channel length, showing the expected linear scaling (R_e_ ∝ *l*) for all CQD sizes. Among the three samples, the smallest CQDs (d~3.06 nm) exhibit the highest resistance, followed by the d~3.78 nm and d~5.27 nm CQDs, respectively. Finally, Figure 3e summarizes the extracted conductivity as a function of CQD size. A clear trend is observed in which larger CQDs exhibit higher conductivity (0.001 S/m), while smaller CQDs show higher resistivity (conductivity: 0.00005 S/m). This trend indicates that films with smaller CQDs show higher resistance, and larger CQDs achieve higher conductivity. We suggest that this behavior is due to two main factors: (1) smaller CQD films have a larger number of particle–particle interfaces, while larger CQDs have more “intra-dot” bulk conductance and less dot-to-dot interfaces, improving conductivity; and (2) larger CQDs with a smaller bandgap result in higher thermal intrinsic carrier generation than for the smallest CQDs (see Supplementary Information Section S4). These two factors support the higher conductivity observed for the larger CQDs; however, further analysis on film morphology, doping, traps, and surface ligands is also required to fully understand the electrical conduction behavior.

**Figure 4:**
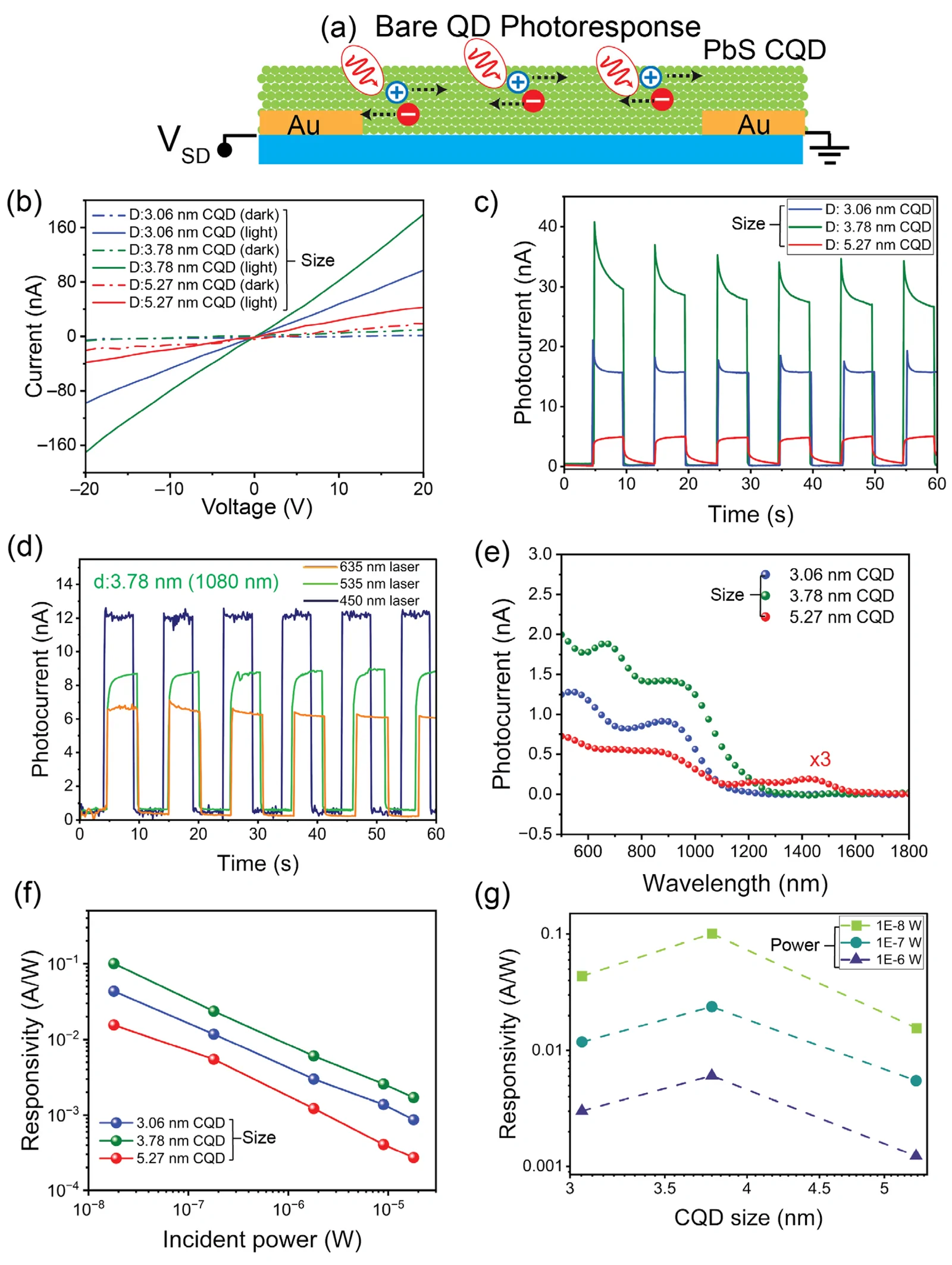
Bare CQD photoresponse. (**a**) Bare CQD photodetection scheme, in which both photocarriers are generated upon illumination and collected at source/drain contacts under a bias voltage. (**b**) I/V curves under dark and light conditions, showing the drastic increase in photocurrent under light. The mid-size d~3.78 nm CQDs show the largest photocurrent. (**c**) Time response under ON/OFF illumination for different CQD sizes under bias voltage of 4 V, confirming the highest response from the d~3.78 nm CQD. (**d**) Time response of d~3.78 nm CQDs for different illumination wavelengths. (**e**) Spectral response for the CQDs, confirming the largest photoresponse for the d~3.78 nm and the lowest response for d~5.27 nm CQDs across a broad range of the spectrum. The spectrum for d~5.27 nm is amplified 3×. (**f**) Responsivity as a function of illumination power, showing a reduced response as light power increases (λ~635 nm). (**g**) Responsivity as a function of CQD size for different powers (λ~635 nm), showing the superior performance for d~3.78 nm CQDs.

Figure 4 shows the light response of the bare CQD films, which characterizes the photoresponsivity and photodetection performance of bare CQD films. The tested devices are the same as in Figure 3, with a CQD film thickness of 100 nm, but under light illumination conditions. Figure 4a shows a schematic of the device photoresponse, which requires the effective transport of both photogenerated carriers (holes and electrons) and is usually limited by the transport of the minority carriers. However, photoresponse is also boosted by traps that may extend carrier lifetime and therefore photogain. Figure 4b shows the I/V curve under dark and light conditions using a 635 nm laser diode. A pronounced increase in current is observed under light conditions compared to dark conditions for all CQD sizes. Among them, the d~3.78 nm (λ_e_~1080 nm) CQDs exhibit the largest photoresponse, followed by the smallest (d~3.06 nm) CQDs, while the largest (d~5.27 nm) CQDs show the lowest photocurrent. The time-resolved ON/OFF response in Figure 4c confirms this trend over repeated illumination cycles. The d~3.78 nm CQDs show the highest photocurrent (I*_ph_*), followed by the d~3.06 nm and then the d~5.27 nm devices. Under 635 nm wavelength illumination at 1 mW, I*_ph_* levels are approximately ~35–40 nA (1080 nm), ~15–20 nA (935 nm), and ~5–7 nA (1550 nm) under a bias voltage of 4 V. Figure 4d shows the response of d~3.78 nm CQDs under different wavelength illuminations, with stronger response for shorter wavelengths probably due to stronger light absorption. The spectral response in Figure 4e shows broadband photodetection from the visible into the near-infrared for all CQD sizes, with the d~3.78 nm CQDs maintaining the highest photocurrent across most of the spectrum, followed by the smallest d~3.06 nm CQDs and then the d~5.27 nm CQDs (amplified 3×), confirming the behavior from Figure 4c, as well as the stronger response for shorter wavelengths. A gradual roll-off at longer wavelengths reflects the band-edge absorption of each CQD size. Figure 4f shows the responsivity as a function of illumination power. The responsivity decreases with increasing incident optical power for all devices, consistent with trap-mediated photoconductive gain [36,37,38]. As expected from the spectral response, the d~3.78 nm CQDs exhibit the highest responsivity, while the d~5.27 nm CQDs show the lowest, with the d~3.06 nm devices in between. These results strongly indicate that despite the higher conductance of the large d~5.27 nm CQDs, they deliver the lowest photoresponse, indicating that higher electrical conductance does not directly imply a high photoresponse performance.

**Figure 5:**
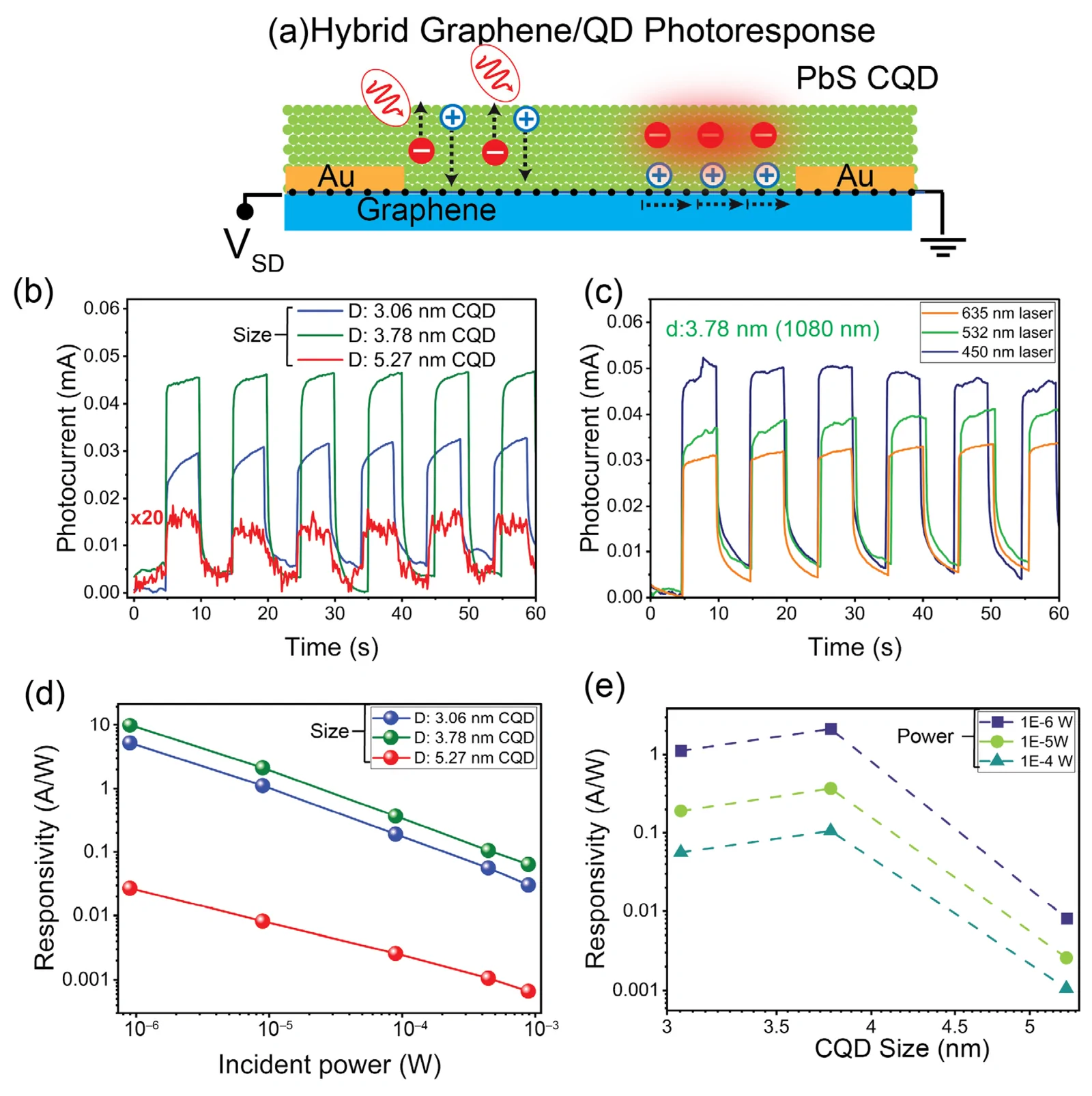
Hybrid graphene/CQD photodetectors. (**a**) Operation of hybrid Gr/CQD photodetectors. Light generates photocarrier photocarriers in the CQDs. Photogenerated holes are then transferred to graphene, increasing the conductivity to produce a positive photocurrent. The photogenerated electrons accumulate in the CQD film, producing a photogain effect. (**b**) ON/OFF time photoresponse under λ~635 nm illumination and bias voltage of 1 V, showing the highest response for the d = 3.78 CQDs, and a drastically inferior response for d = 5.27 CQDs (trace scaled up 20×). (**c**) ON/OFF time photoresponse for the d~3.78 CQDs for different light wavelengths. (**d**) Responsivity as a function of light power for λ~635 nm, showing a drastically weaker response for d~5.27 across a wide range of powers. (**e**) Responsivity for Gr/CQD photodetectors as a function of CQD size at various light powers. The larger d~5.27 nm CQDs show a significantly lower responsivity.

Finally, Figure 5 shows our results studying the performance of ~100 nm thick CQD films in a hybrid Gr/CQD photodetection scheme, as shown in Figure 5a. In this case, the CQDs absorb light and generate photocarriers. Due to the band alignment between graphene and PbS CQDs, photogenerated holes in the CQDs are transferred to graphene, increasing its conductivity, whereas photoelectrons remain in the CQD film, producing a photogating effect [10]. In this configuration, a critical aspect is the transport of photogenerated holes to the graphene layers. In addition, the photogenerated electrons accumulate in the CQD film, producing a photogain effect. Figure 5b shows the time response under ON/OFF illumination and a bias voltage of 1 V. The d~3.78 nm (λ_e_~1080 nm) CQDs still show a superior performance in the Gr/CQD configuration, followed closely by the d~3.06 nm CQDs (λ_e_~935 nm). Notably, the d~5.27 nm CQDs (λ_e_~1550 nm) show by far the lowest photoresponse. Its trace is multiplied by a factor of 20 in Figure 5b. Then, Figure 5c shows the time response of the d~3.78 nm CQDs under different excitation wavelengths (450 nm, 532 nm, and 635 nm), demonstrating a systematic trend in which the photocurrent decreases as the illumination wavelength increases. This behavior is consistent with reduced absorption at longer wavelengths, confirming the expected spectral dependence of the device observed also for bare CQDs in Figure 4e. The responsivity trends in Figure 5d show the expected decrease in responsivity with increasing incident optical power for all CQD sizes, indicative of trap-limited photoconductive gain, which has been widely reported for such hybrid Gr/CQD systems [10,29]. Importantly, the 1550 nm CQDs consistently exhibit the lowest responsivity across the entire power range. At 10^−6^ W, the d~3.78 nm CQDs reach responsivity close to ~10 A/W, while the d~5.27 nm CQDs reach 3 × 10^−2^ A/W, a significant reduction close to two orders of magnitude. Finally, Figure 5e summarizes the responsivity for different illumination powers as a function of CQD size. The d~3.78 nm CQDs exhibit the highest responsivity, followed by similar responsivity of the d~3.06 CQDs, and then by a drastic performance reduction of nearly two orders of magnitude of the largest d~5.27 nm CQDs. To explore the potential impact of band alignment variations due to QD size, we performed ultraviolet photoelectron spectroscopy (UPS) on CQD films and transconductance measurements on a hybrid Gr/QD film, as shown in Supplementary Information Section S5. From UPS, for QD sizes of d~3.06 nm, ~3.78 nm and ~5.27 nm, the corresponding work functions **Φ** are ~5.321, ~5.157 and ~5.308 eV, showing small variations that do not correlate with photoresponse behavior. Similarly, transconductance measurements of hybrid Gr/QD films show that the Fermi level with respect to the Dirac point for CQD sizes of d~3.06 nm, ~3.78, and ~5.27 nm size correspond to ~0.174 eV, ~0.207 eV, and ~0.205 eV. These variations are small and do not correlate with photoresponse either. From UPS and transconductance analysis on Fermi levels for different CQD sizes, we suggest that the band alignment by itself cannot explain the experimentally observed behavior of significantly lower response for larger CQDs. It is important to remark that previous reports also showed a reduction in nearly two orders of magnitude in photoresponse of Gr/CQD systems from small CQDs to large CQDs [10,31], in agreement with our results reported in Figure 5.

## 4. Discussion

The major finding from this work is that despite the higher conductivity of the large d~5.27 nm CQDs, this does not result in a strong photodetection performance for the architectures presented here. The larger conductivity for larger CQD size has been observed and discussed in previous reports [34], resulting from the lower electron charge wavefunction confinement, stronger delocalization, fewer particle-to-particle hops, more intra-dot bulk conduction, and large carrier concentration due to smaller bandgap, with all these factors increasing the charge mobility and concentration [39,40,41]. Temperature-dependent I–V measurements under applied bias could be a route to distinguish ohmic, space-charge-limited, and trap-controlled transport, thereby clarifying the roles of carrier injection and trap states in electrical conductivity [42,43]. However, the lower photoresponse of the largest CQDs compared to the smaller CQDs illustrates that higher conductivity does not translate directly into improved photodetection. It is important to remark that the three CQD films show similar absorption coefficients (6 × 10^4^ 1/cm at λ~635 nm, Supplementary Information Section S3) and have a thickness of ~100 nm. Therefore, the lower response for large d~5.27 nm CQDs cannot be due to weaker light absorption, as the light absorption coefficient and film thickness are similar for the three types of CQDs. In the case of the hybrid Gr/CQD configuration, a similar trend of lower photoresponse for larger CQDs has been observed. In fact, in one of the first reports on hybrid Gr/CQD photodetectors from Konstantatos et al. (2012) [10], CQDs with exciton peak at λ_e_~900 nm reach responsivities of 6 × 10^7^ A/W, while CQDs with λ_e_~1450 nm reach lower responsivities of 1 × 10^6^ A/W, similar to our results. Ahn et al. also observed larger photoresponse for smaller CQDs in hybrid Gr/CQD configurations [31]. However, we want to point out that given the large effect that trap-mediated photoconductive gain plays in CQD photodetection [36], it is possible that the large number of interfaces in small CQD films (larger surface/volume ratio) leads to significantly larger trapping times, resulting in higher photogains and therefore significantly increasing the photoresponse for smaller CQDs [28,36]. A similar effect may play a role in the bare CQD photodetection results, since trap-mediated photogain mechanisms are also present in bare CQD photodetectors [28]. However, it is important to mention that in another report, Huang et al. (2023) found that larger PbS CQDs give larger photoresponse for bare CQD photodetectors [43]. However, they use different deposition conditions, including a different solvent (octane) and heavier CQD concentrations of 50 mg/mL, compared to our concentrations that are less than 20 mg/mL. These differences can lead to different film morphologies and, therefore, optoelectronic performance. This clearly indicates that more studies will be required to establish a definitive trend and mechanisms regarding the effect of CQD size on optoelectronic device performance. However, our experimental data show that CQD size does have a strong impact on photodetection performance. Further experiments would require analysis of other factors such as film morphology, surface ligands and passivation, temperature transport dependence, recombination processes, as well as more detailed studies on band alignment between CQDs and graphene, to clarify the mechanisms by which CQD size affects their photoresponse. A solid explanation for this behavior requires further analysis and consideration of other factors. There are also significant advances in alternative passivation/heterojunction architecture strategies that can affect CQD surface states and oxidation that can be used to control and enhance charge separation and transfer in PbS hybrid structures [27]. In summary, we think that any size tuning of the CQD should be accompanied by a detailed analysis of its impact on the device performance as well as consideration of alternative architectures to control traps and charge transfer.

## 5. Conclusions

This work demonstrates that beyond the spectral response, CQD size also plays a central role in determining the electrical conductivity and photoresponsivity of PbS CQD-based photodetectors. While larger CQDs exhibit enhanced electrical conductivity mainly due to reduced dot-to-dot hopping, they do not yield superior photodetection performance. Instead, smaller CQDs with sizes of ~3.06 nm and ~3.78 nm show higher photoresponse in both bare CQD films and hybrid Gr/CQD devices. This result highlights that higher conductivity does not translate directly into larger photoresponse. Further experiments are required to fully explain this behavior, especially by analyzing critical aspects such as film morphology, surface ligands and passivation, recombination processes, thermal effects, as well as band alignment. We also discussed the possibility that the increased photodetection performance of smaller CQDs can be attributed to factors such as increased traps leading to longer lifetime and photogain. Our results also indicate that across all CQD sizes and for bare and Gr/CQD detectors, responsivity decreases with increasing optical power, confirming the role of trap-mediated photoconductive gain. The results observed in both device architectures establish a clear necessity to analyze the impact of CQD size and spectral tuning to avoid a drastic drop in photodetection performance. These findings provide important considerations for the design of next-generation CQD-based optoelectronic devices, particularly in applications requiring broadband and tunable photodetection with high responsivity.

## Data Availability

The original contributions presented in this study are included in the article/Supplementary Materials. Further inquiries can be directed to the corresponding authors.

## Supplementary Materials

The following supporting information can be downloaded at: https://www.mdpi.com/article/10.3390/nano16171073/s1, Section S1: CQD Size Analysis; Section S2: Atomic Force Microscopy Analysis; Section S3: Extended UV/Vis absorption of CQDs from 500 nm to 1800 nm; Section S4: Effect of bandgap on intrinsic carrier concentration; Section S5: UPS and Transconductance.

## Abbreviations

The following abbreviations are used in this manuscript:

CQD	Colloidal Quantum Dots
TBAI	Tetrabutylammonium iodide
Gr	Graphene
TEM	Transmission Electron Microscope
SEM	Scanning Electron Microscope
PMMA	Poly(methyl methacrylate)
t	Thickness
L	Number of layers
t_L_	Thickness per layer
*l*	Channel length
α	Absorption coefficient
λ	Wavelength
λ_e_	Exciton peak wavelength
I*_ph_*	Photocurrent
