# Supplementary material for: Evaluating the Impact of the Size of PbS Colloidal Quantum Dots on Photodetection Performance
Source: Nanomaterials (Basel). 2026 Aug 28;16(17):1073. doi: 10.3390/nano16171073 (PMC13567662; doi:10.3390/nano16171073)
Supplement: Supplementary file 1 [file nanomaterials-16-01073-s001.zip › nanomaterials-4418249-supplementary.pdf]

Evaluating the Impact of the Size of PbS Colloidal Quantum Dots on Photodetection Performance

Freddy Garcia [1], Suraj Patel [1,2], Anthony Monterrosas [1], John Navarro [1], Seungbae Ahn [3,*] and Oscar Vazquez-Mena [1,2,*]

[1] Aiiso Yufeng Li Family Department of Chemical and Nano Engineering, Center for Memory and Recording Research, University of California San Diego, La Jolla, CA 92093, USA; f8garcia@ucsd.edu (F.G.); sup027@ucsd.edu (S.P.); amonterrosasmendoza@ucsd.edu (A.M.); jon016@ucsd.edu (J.N.)

[2] Materials Science and Engineering Program, University of California San Diego, La Jolla, CA 92093, USA

[3] Division of Electronics and Electrical Information Engineering, Korea Maritime and Ocean University, Busan 49112, Republic of Korea

* Correspondence: sbahn@kmou.ac.kr (S.A.); ovmena@ucsd.edu (O.V.-M.)

Section S1: CQD Size Analysis

Section S2: Atomic Force Microscopy Analysis

Section S3: Extended UV/Vis absorption of CQDs from 500 nm to 1800 nm

Section S4: Effect of bandgap on intrinsic carrier concentration

Section S5: UPS and Transconductance

Section S1: CQD Size and crystalline analysis.

Histograms were obtained by analyzing 10 HRTEM micrographs with CQDs.

Exciton Peak	Representative TEM images	Histogram	Size Analysis<br>X: Mean<br>σ: St Dev
935 nm	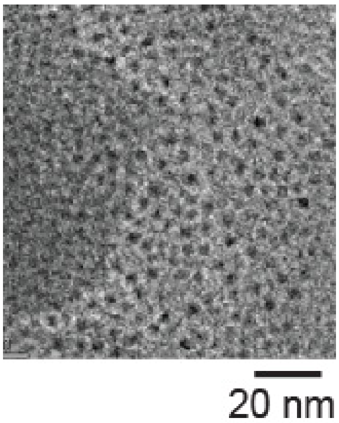	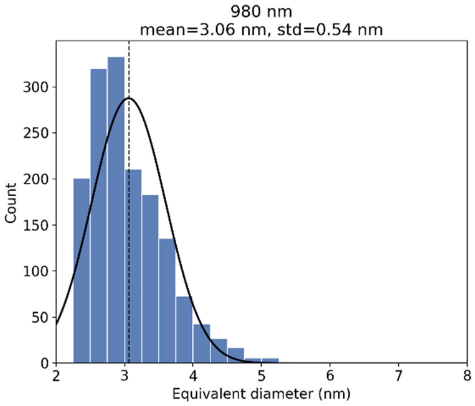	X = 3.06 nm<br>σ = 0.54 nm
1080 nm	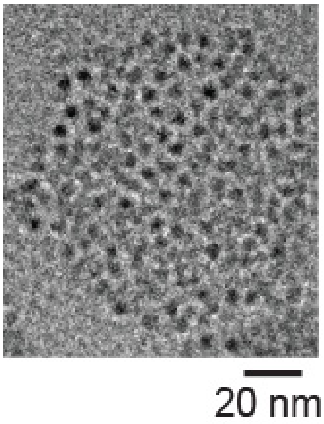	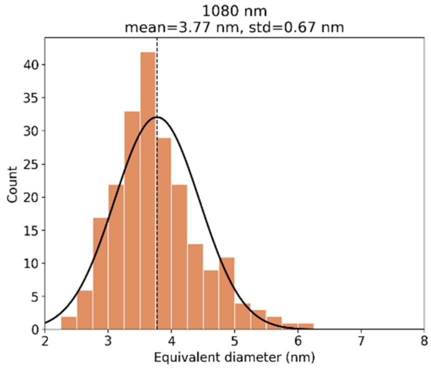	X = 3.78 nm<br>σ = 0.67 nm
1550 nm	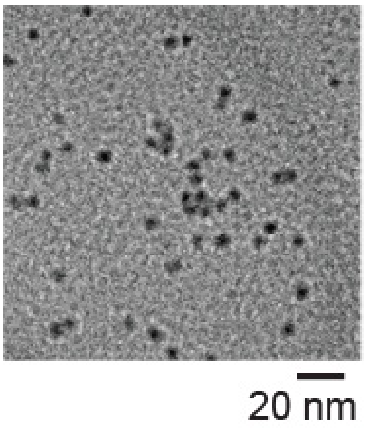	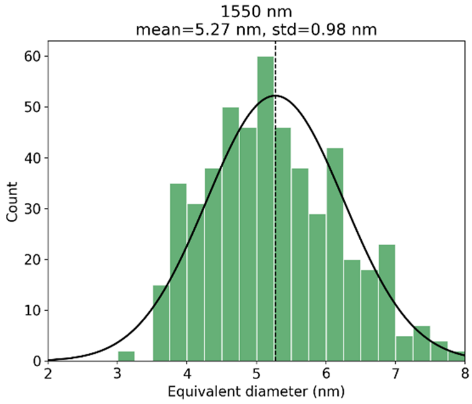	X = 5.27 nm<br>σ = 0.98 nm

Digital FFT analysis.

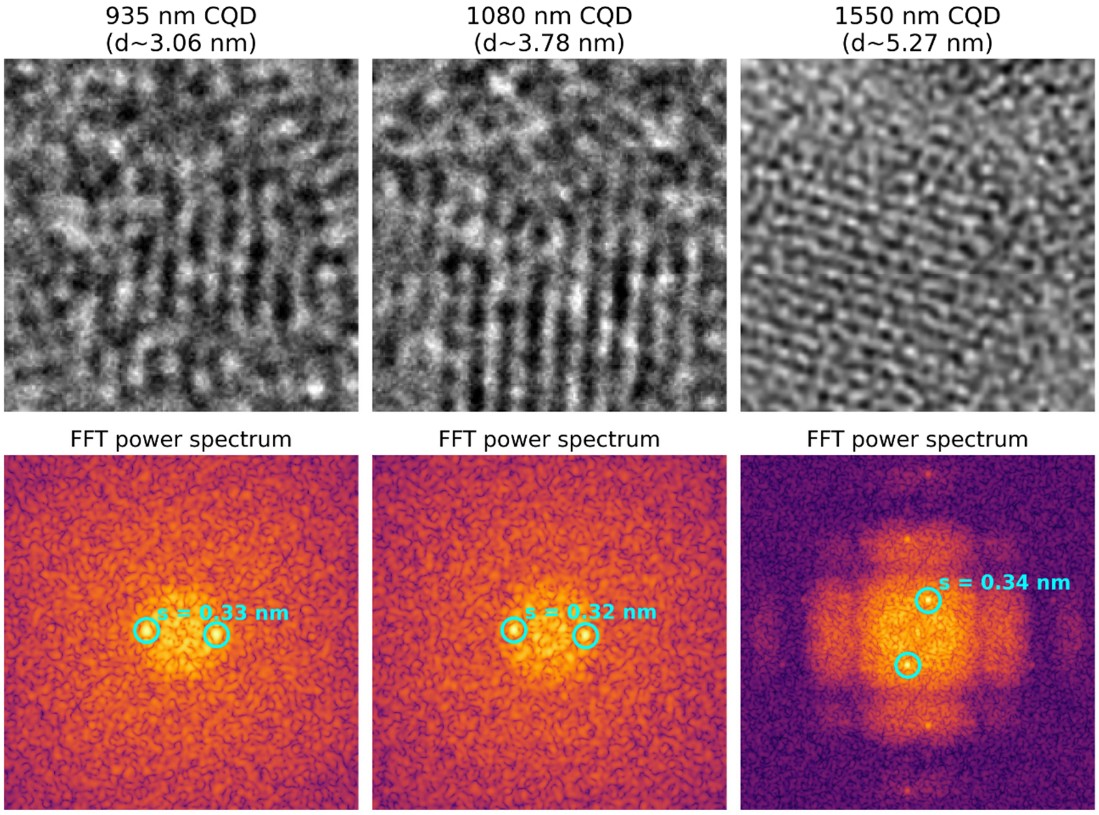

Each panel shows one sharp, symmetric spot pair in the FFT (Friedel pair — mirrored through the center, same intensity, same spacing). That's the diagnostic signature of a single set of periodic lattice planes, i.e., one crystalline domain per particle. No diffuse halo that would indicate amorphous material, nor multiple spot pairs at different angles or spotty rings (which would indicate polycrystalline). Measured fringe spacings ('s') are: s=0.33 nm for d:3.06 nm, s=0.32 nm for d:3.78 nm, and s=0.34 nm for d:5.27 nm— internally consistent across the three particles, which supports these being genuine lattice fringes rather than noise.

Section S2: Atomic Force Microscopy Analysis

AFM analysis. QD Size: 3.06 nm. Roughness: 4.755 nm

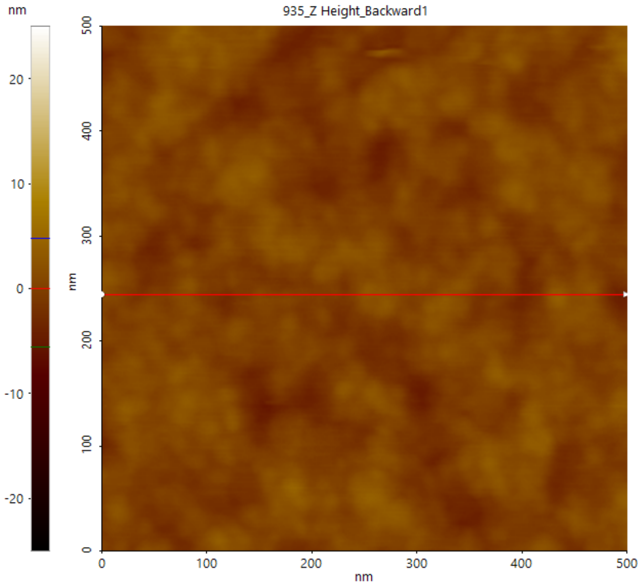

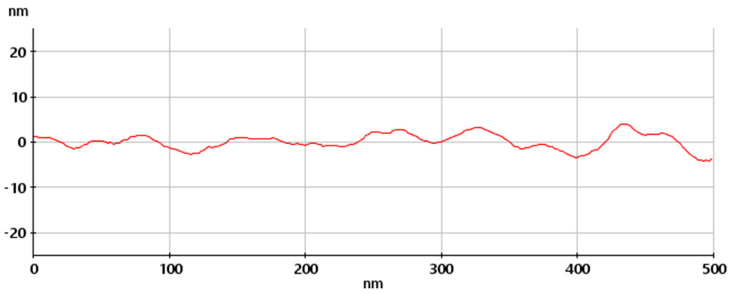

Line profile from red trace in scan image (above).

Given the thickness of 100 nm of the films, a 4.7 nm roughness represents 4.7% of the thickness. The roughness is roughly equivalent to the size of a single CQD. The film does not show pin-holes or voids that compromise the integrity and uniformity of the films.

AFM analysis. QD Size: 3.78 nm. Roughness: 4.311 nm

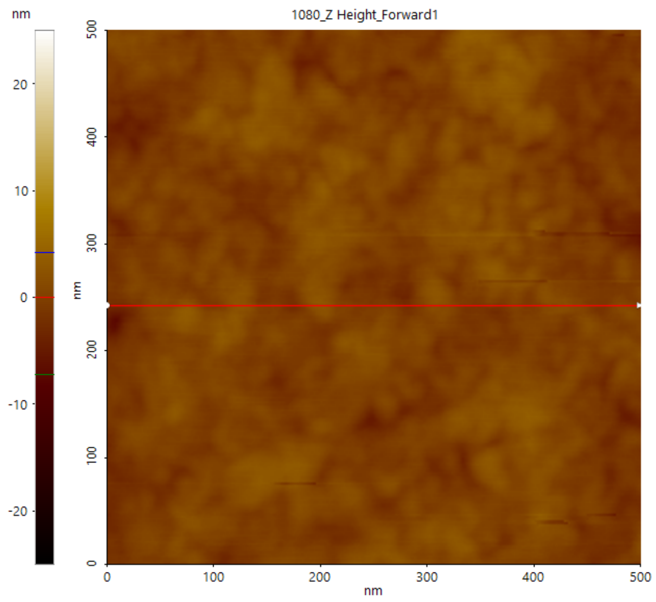

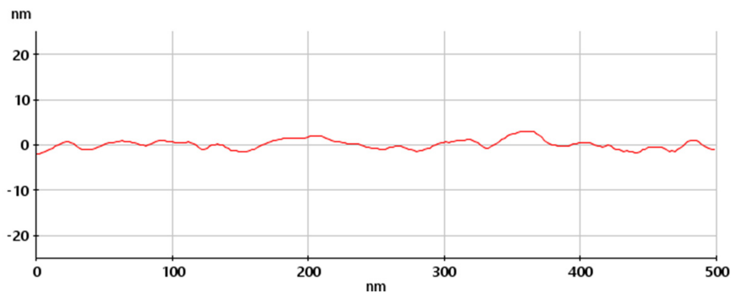

Line profiles from red trace in scan image (above).

Given the thickness of 100 nm of the films, a 4.3 nm roughness represents 4.3% of the thickness. This roughness is roughly equivalent to the size of a single CQD. The film does not show pin-holes or voids that compromise the integrity and uniformity of the films.

AFM analysis. QD Size: 5.27 nm. Roughness: 7.895 nm

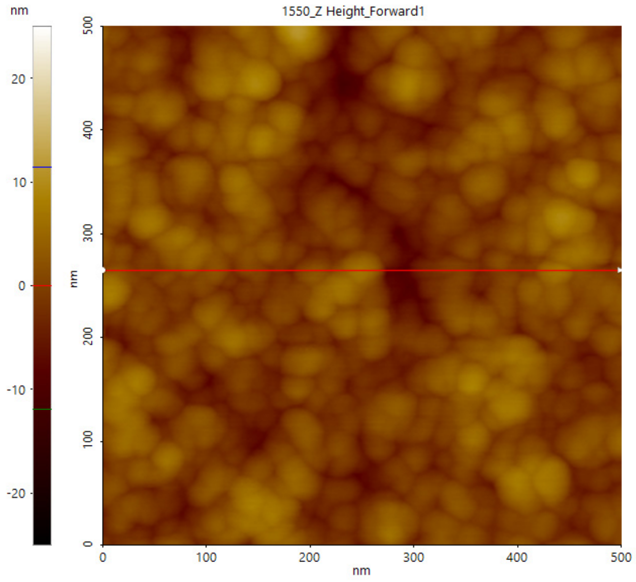

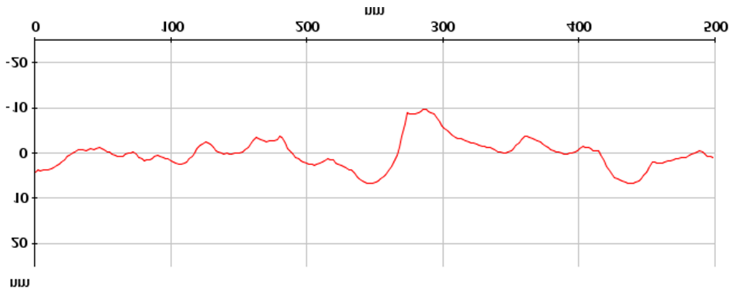

Line profile from red trace in scan image (above).

Given the thickness of 100 nm of the films, a 7.8 nm roughness represents 7.8% of the thickness. This roughness is slight larger than the size of a single CQD (5.27 m). The film does not show pin-holes or voids that compromise the integrity and uniformity of the films.

Section S3: Extended UV/Vis absorption of CQDs from 500 nm to 1800 nm

Absorption coefficient from 500 nm to 1800 nm
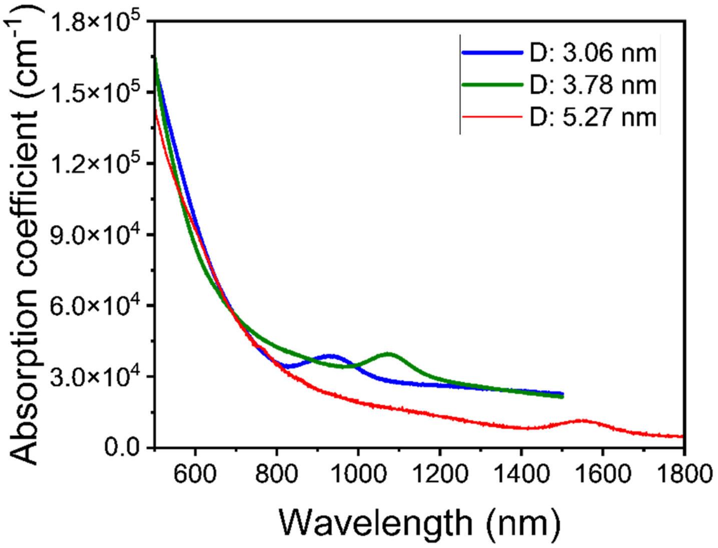

Section S4: Effect of bandgap on intrinsic carrier concentration

**Comparison of intrinsic carrier concentration at room temperature (300 K) due to different bandgaps for QD of different sizes. We assume similar density of states with difference dominated by Boltzmann factor $exp\left(\frac{Eg}{2k_BT}\right)$.**			
QD Size	Bandgap (E_g)	Bandgap Difference E_g(QD1) – E_g(QD1)	Carrier concentration (ni) with respect to smallest QD1 (3.06 nm)
$\frac{n_i(QD2)}{n_i\,(QD1)} = exp\left(\frac{E_g\,(QD2)-E_g\,(QD1)}{2k_BT}\right)$, $\frac{n_i(QD3)}{n_i\,(QD1)} = exp\left(\frac{E_g\,(QD2)-E_g\,(QD1)}{2k_BT}\right)$			
QD1 Size~3.06 nm	1.08 eV	0	$\boldsymbol{\frac{n_i(QD2)}{n_i\,(QD1)} = 1}$ **(Reference value)**
QD2 Size~3.78 nm	0.98 eV	0.1	$\boldsymbol{\frac{n_i(QD2)}{n_i\,(QD1)} \approx 7}$
QD3 Size~5.27 nm	0.77 eV	0.31	$\boldsymbol{\frac{n_i(QD2)}{n_i\,(QD1)} \approx 400}$

Section S5: UPS and Transconductance

UPS measurements: The spectra were collected using He-I excitation (hν=21.22 eV). The work function was calculated using the cutoff from the secondary electrons (SECO),

$\Phi = h\nu - (E_{cutoff})$.

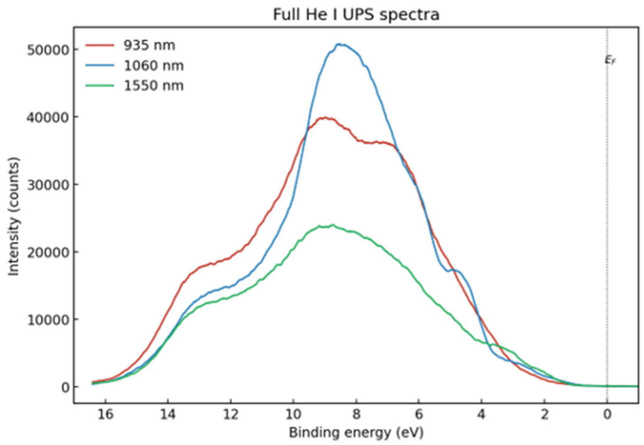

The work function for the 100 nm thick QD films gave the following work-functions and R2 fitting values.

Sample	Work function Φ	Fitting: R2 (SECO)
d: 3.06 nm	5.321 eV	0.994
d: 3.78 nm	5.157 eV	0.991
d: 5.27 nm	5.308 eV	0.988

Transfer curves (I_{ds} vs V_g) for QD/Gr systems:

Transfer curves for graphene layers with 100 nm thick QDs on top. The curves therefore represent transfer curves with the Fermi levels between the QDs and Gr already aligned, giving us an indication on how the bands align between graphene and QDs for different QD sizes. The minimum curves represent the charge neutrality point (V_{CNP}), which is the gate voltage required to reach the Dirac point.

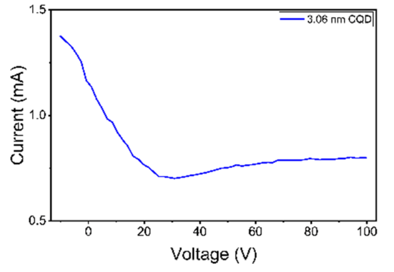	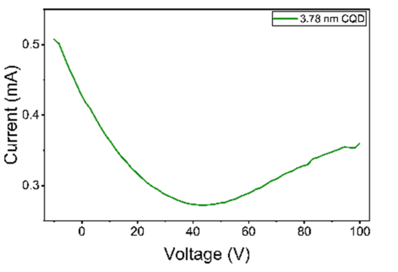	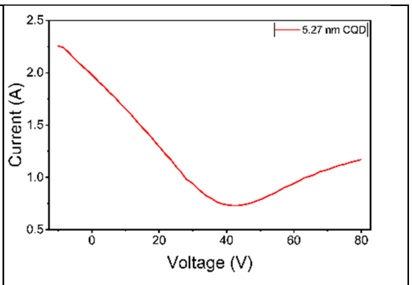
V_{CNP}~ 31 V	V_{CNP}~ 44 V	V_{CNP}~ 43 V

Using a 300 nm SiO_2 back-gate dielectric, ε_r=3.9, and v_F=1.05×10^6 m/s and the following equations,

$$n = \frac{C_{ox}V_g}{e}$$

$$E_F - E_D = \hbar v_F \sqrt{\pi n}$$

We can extract the position of the Fermi level with respect to the Dirac point.

QD	V_{CNP}	Carrier density @ Vg=0	Carrier type @ Vg=0	**E_F below Dirac Point**
935 nm	31 V	2.23×10^{6v} cm^{-2}	Holes	**0.174 eV**
1080 nm	44 V	3.16×10^{6} cm^{-2}	Holes	**0.207 eV**
1550 nm	43 V	3.09×10^{6} cm^{-2}	Holes	**0.205 eV**
